# Tumor Budding in Colorectal Cancer: Biological Basis, Clinical Risk Stratification, and Digital Pathology

**DOI:** 10.3390/cancers18213493

**Published:** 2026-10-29

**Authors:** Enrique Bernal-Morell, Francisco Martínez-Díaz, Francisco García-Molina

**Affiliations:** 1 Department of Internal Medicine, Hospital General Universitario Reina Sofía, 30003 Murcia, Spain; ebm.hgurs@gmail.com; 2 Department of Medicine, Faculty of Medicine, University of Murcia, 30120 El Palmar, Spain; 3 Department of Pathology, Hospital General Universitario Reina Sofía, 30003 Murcia, Spain; fmdiaz@um.es; 4 Department of Ophthalmology, Optometry, Otolaryngology and Pathology, Faculty of Medicine, University of Murcia, 30120 El Palmar, Spain

**Keywords:** tumor budding, colorectal cancer, ITBCC, epithelial–mesenchymal transition, prognosis, digital pathology, deep learning

## Abstract

Tumor budding, defined as single tumor cells or clusters of up to four cells at the invasive front, is a morphological marker of tumor-cell dissociation and stromal invasion in colorectal cancer. Standardization by the International Tumor Budding Consensus Conference (ITBCC) established a reproducible hematoxylin-and-eosin-based method and enabled broader incorporation of budding into pathology reporting and clinical risk assessment. Intermediate- and high-grade budding are associated with lymph node metastasis in pT1 colorectal cancer, while high-grade budding identifies a subgroup of stage II colon cancers with increased recurrence risk. Adverse prognostic associations have also been reported in stage III disease and in rectal cancer after neoadjuvant therapy, although evidence that budding predicts benefit from a specific treatment is still insufficient. At the biological level, budding is linked to partial epithelial–mesenchymal transition, altered cell adhesion, cytoskeletal remodeling, and interactions with fibroblasts, extracellular matrix, and immune cells. Digital pathology and artificial intelligence may improve hotspot selection, quantification, and spatial characterization, but their clinical use depends on external validation, control of domain shift, and continued pathologist oversight. Tumor budding is therefore best interpreted as a complementary morphological biomarker alongside TNM stage, established histopathological risk factors, molecular features, and clinical context.

## Simple Summary

Tumor budding refers to single cancer cells or small clusters of up to four cells that detach from the main colorectal tumor and invade the surrounding tissue. It is associated with lymph node metastasis, recurrence, and poorer survival. Its clearest current clinical applications are risk assessment after endoscopic resection of pT1 colorectal cancer and prognostic stratification in stage II colon cancer. This review summarizes the definition and assessment of tumor budding, examines the biological mechanisms underlying this invasive phenotype, evaluates its clinical relevance, and discusses how digital pathology and artificial intelligence may improve reproducibility and support future risk models.

## 1. Introduction: Why Additional Prognostic Markers Are Needed in Colorectal Cancer

Colorectal cancer remains a major cause of cancer-related morbidity and mortality. Screening, improved surgery, optimized systemic therapy, and molecular biomarkers have substantially changed its management, yet individual risk prediction remains imperfect. TNM stage is the foundation of prognostic assessment, but patients with apparently similar tumors can experience markedly different clinical courses. This uncertainty is particularly relevant in pT1 disease, where surveillance must be weighed against additional oncologic resection after endoscopic removal, and in stage II colon cancer, where the potential benefit of adjuvant chemotherapy must be balanced against toxicity and competing risks.

Tumor budding is among the most extensively studied histopathological markers of aggressive behavior in colorectal cancer. It reflects tumor-cell dissociation at the invasive front and is associated with stromal invasion and metastatic competence. Accordingly, tumor budding is best regarded as a morphological correlate of an invasive phenotype rather than an isolated microscopic finding [1,2,3]. A recent overview also traces the development of tumor budding from a descriptive histological feature to a clinically relevant prognostic marker [4].

Across multiple cohorts, tumor budding has been associated with lymphovascular invasion, lymph node involvement, recurrence, distant metastasis, and reduced survival. In a retrospective cohort of 192 patients, for example, budding correlated with several adverse clinicopathological variables and with five-year survival [5]. Comparable findings have been reported in locally advanced disease, although recent series differ in case selection, design, and degree of external validation, which should be considered when interpreting their estimates [6].

The strongest evidence for immediate clinical relevance concerns pT1 colorectal cancer and stage II colon cancer. In pT1 disease, budding contributes to the estimation of lymph node metastasis risk after endoscopic resection. In stage II colon cancer, high-grade budding identifies patients at increased risk of recurrence and can inform, but should not independently determine, discussions of adjuvant therapy. The SEOM-GEMCAD-TTD guidelines include tumor budding among adverse pathological features, together with T4 category, inadequate lymph node sampling, lymphovascular or perineural invasion, obstruction, perforation, and poor differentiation [7].

This narrative review integrates consensus recommendations, clinical guidelines, systematic reviews and meta-analyses, large observational cohorts, and representative mechanistic studies. The emphasis is on evidence that informs standardized assessment, biological interpretation, prognosis, clinical decision-making, and emerging computational applications. The review focuses on clinically relevant findings, areas of consistent evidence, and questions that remain methodologically or biologically unresolved.

The literature search supporting this narrative review was conducted in PubMed/MEDLINE using combinations of the terms “tumor budding” and “colorectal cancer”, with primary emphasis on publications from January 2021 to September 2026. Original research articles, review articles, consensus documents, clinical guidelines, and other relevant publications addressing the pathological, prognostic, molecular, and clinical significance of tumor budding were considered. Selected earlier publications were also included when they represented landmark studies, internationally accepted recommendations, or particularly relevant contributions to the definition, assessment, and clinical interpretation of tumor budding. Given the narrative nature of this review, no formal systematic-review methodology was applied.

Compared with recent narrative reviews by Ciobănoiu and colleagues [4] and Zhang and colleagues [8], the present review places particular emphasis on the strength and limitations of evidence across clinical decision settings, the distinction between prognostic and treatment-predictive utility, and the integration of ITBCC morphology with spatial molecular heterogeneity and computational pathology. The additional discussion of CMS2-to-CMS4-like invasive-front plasticity is intended to connect mechanistic findings with practical interpretation, without implying that molecular subtype can be assigned from budding morphology alone.

## 2. Histopathological Definition of Tumor Budding

Tumor budding is defined as a single tumor cell or a cluster of up to four tumor cells separated from the main tumor mass, usually at the invasive front of colorectal carcinoma [1]. Morphologically, budding marks the transition from cohesive gland formation to small, dissociated cellular units in direct contact with the surrounding stroma, extracellular matrix, vessels, fibroblasts, and immune cells.

Peritumoral budding is assessed at the invasive front of endoscopic or surgical resection specimens. This is the setting in which the International Tumor Budding Consensus Conference (ITBCC) method was validated and for which the prognostic evidence is strongest [1]. Intratumoral budding occurs within the tumor body and may be relevant in biopsies that do not sample the invasive front. It can provide preoperative information, but its assessment and clinical cutoffs are not yet standardized to the same degree [9,10].

Tumor budding is distinct from histologic grade. Grade reflects gland formation and the overall architectural differentiation of the tumor, whereas budding measures cellular dissociation and stromal invasion. A moderately differentiated adenocarcinoma may therefore display extensive budding and behave more aggressively than its conventional grade would suggest.

Tumor buds must also be distinguished from poorly differentiated clusters (PDCs). A bud contains one to four tumor cells, whereas a PDC contains five or more tumor cells without gland formation. Although this numerical threshold is operational, emerging spatial and molecular data support a biological continuum of tumor-cell dissociation in which progressively smaller clusters may display increasingly invasive features [11].

Tumor budding is not unique to colorectal cancer. Intermediate- or high-grade budding has been associated with adverse outcomes in intestinal-type sinonasal adenocarcinoma [12] and intestinal-type gastric adenocarcinoma [13]. These small observational studies suggest that budding may also characterize epithelial invasion outside colorectal cancer; however, the gastric association with survival was not independently significant in multivariable analysis. Nevertheless, scoring systems, cutoffs, and clinical implications remain organ-specific and should not be transferred directly from colorectal cancer to other tumor types.

## 3. Diagnostic Standardization: The ITBCC Method and Practical Challenges

Standardization was essential to translate tumor budding from a variably reported observation into a clinically useful biomarker. Earlier studies differed in staining method, field area, hotspot selection, cutoffs, and grading systems. The 2016 ITBCC established a common approach based on hematoxylin and eosin (H&E), selection of the area with the highest bud density at the invasive front, and counting within a field normalized to 0.785 mm^2^ [1].

The ITBCC categories are Bd1 (0–4 buds), Bd2 (5–9 buds), and Bd3 (≥10 buds). Because budding is a continuous variable, the absolute bud count should be reported together with the categorical grade, particularly in cases close to a cutoff. A concise formulation is: “Tumor budding assessed according to ITBCC: X buds per 0.785 mm^2^; Bd1/Bd2/Bd3”.

H&E is the ITBCC reference stain. Pancytokeratin immunohistochemistry may assist in difficult cases, especially when inflammation or complex stroma obscures isolated epithelial cells. However, cytokeratin staining may also increase apparent bud counts by highlighting epithelial fragments that do not fulfill morphological criteria for true budding. H&E- and cytokeratin-based counts should therefore not be considered analytically interchangeable, and immunohistochemistry is best used as an adjunct rather than a substitute for morphological assessment.

Pseudobudding is an important diagnostic pitfall. Small cell groups surrounding ruptured glands in heavily inflamed areas may resemble tumor buds but represent a reactive process rather than active invasion. Overcalling these structures may inflate the bud count and alter risk classification [14].

Digital pathology offers a potential route to more reproducible hotspot selection and quantification, especially in clinical trials, multicenter studies, and high-volume diagnostic practice. At present, its most defensible role is as decision support: automated detections still need pathological review to distinguish true buds from pseudobudding, poorly differentiated clusters, and technical artifacts [3].

The main elements of ITBCC-based tumor budding assessment and the most relevant diagnostic pitfalls are summarized in Table 1.

## 4. Biological Basis: Partial EMT, Adhesion, and Tumor-Cell Plasticity

Tumor budding is unlikely to arise from a single molecular pathway. Current evidence instead supports a convergent phenotype involving reduced cell–cell adhesion, phenotypic plasticity, partial epithelial–mesenchymal transition (EMT), extracellular matrix remodeling, stromal interactions, and immune modulation [2].

High-grade budding may also include cytoplasmic pseudofragments continuous with budding cells and detectable by cytokeratin staining. These structures have been associated with aberrant β-catenin, laminin-5 γ2 expression, absence of peritumoral lymphocytic infiltration, and an infiltrative growth pattern, suggesting a particularly active and motile budding phenotype [15].

The “go or grow” model offers one functional interpretation of this phenotype. Buds often display low proliferative activity and low apoptosis, suggesting partial segregation between migratory and proliferative states. The model is biologically plausible, but it should not be taken to imply that all buds are quiescent or that proliferation and migration are mutually exclusive [2].

Loss of CDX2 provides mechanistic support for a link between dedifferentiation and invasion. Buds show lower CDX2 and E-cadherin expression than the main tumor. In experimental models, CRISPR/Cas9-mediated CDX2 loss increases migration, whereas restoration of CDX2 partly rescues the phenotype [16].

At the level of molecular interpretation, the relevant question is not whether budding represents complete epithelial-to-mesenchymal transition (EMT), but whether localized epithelial plasticity is sufficient to facilitate detachment and invasion. Expression profiling of microdissected budding regions has suggested enrichment of cell-migration and survival programs and attenuation of proliferation-related transcripts relative to the tumor bulk. These observations support a spatially restricted, EMT-like program, rather than an obligatory, irreversible conversion of all budding cells to a fully mesenchymal lineage [17].

The evidence nevertheless has important boundaries. Microdissection may recover admixtures of carcinoma cells and adjacent stroma, and transcriptional programs inferred from small tumor regions do not establish the temporal sequence of detachment, migration, and metastatic colonization. Similarly, the CDX2-loss experiments discussed above provide functional support for enhanced migration, whereas immunohistochemical correlations with SLUG and P4HA2 remain associative. Taken together, the studies implicate cooperating cellular programs rather than a single validated budding-specific driver [16,17,18].

Several candidate molecules fit this invasive model, although the strength of evidence varies. SLUG and P4HA2 expression have been associated with lymphovascular and perineural invasion, extensive budding, and adverse outcome, particularly in KRAS-mutant tumors [18]. These findings provide mechanistic clues, but neither marker is yet supported by the level of independent clinical validation required for routine use.

## 5. Cell Adhesion, Cytoskeletal Reorganization, and Migration

Bud formation requires coordinated changes in cell junctions, extracellular matrix, and cytoskeletal organization. Tumor cells must loosen their dependence on glandular architecture, generate protrusions, and move through a changing stromal environment. Thus, budding represents an active invasive phenotype rather than passive cellular detachment.

The CDH17-DSC1-p120-catenin complex illustrates context-dependent reorganization of cell adhesion. CDH17 interacts with desmocollin-1 and p120-catenin and modulates migration and invasion. DSC1 expression in budding cells at the leading edge, together with its adverse prognostic association in stem-like and mesenchymal colorectal cancer, suggests that adhesion molecules may facilitate invasive plasticity as well as epithelial cohesion [19].

Fascin1, an actin-bundling protein involved in cellular protrusions, provides a possible therapeutic connection. The published HITCLIF phase II trial protocol describes an evaluation of imipramine as an anti-invasive strategy in Fascin1-overexpressing tumors, including colon and rectal cancers. Histopathological endpoints include changes at the invasive margin, such as budding, cytoplasmic pseudofragments, growth pattern, and peritumoral lymphocytic infiltration [20]. Whether a change in budding can serve as a reliable pharmacodynamic endpoint remains uncertain and will require correlation with clinically meaningful outcomes.

Innate inflammatory signaling may also contribute to the budding phenotype. Combined TLR4/MyD88 expression has been associated with lymph node metastasis and high-grade budding, while selected genetic variants correlate with other adverse features [21]. The pathway is not specific to budding, but it may participate in shaping a microenvironment permissive for invasion.

In a separate observational study, pSTAT3 and CTLA4 overexpression correlated with distant metastasis, higher grade, mitotic activity, extensive budding, an infiltrative border, and poorer overall survival [22]. These associations are consistent with links among pro-tumor signaling, immune escape, and invasion, but the observational design cannot establish that either pathway directly causes budding.

The TLR4/MyD88 and pSTAT3/CTLA4 studies add a signaling and immune dimension, but broad immunohistochemical associations may be confounded by stage, differentiation, stromal abundance, and inflammatory context. These data should not be interpreted as evidence that inhibiting either pathway will necessarily reduce tumor budding or improve survival [21,22]. A mechanistically informative next step would be to spatially map these pathways in true buds, matched bulk tumor, and adjacent stromal compartments and then test their function experimentally.

## 6. Tumor Microenvironment: Stroma, Cancer-Associated Fibroblasts, Extracellular Matrix, and Immunity

Budding cannot be explained solely by tumor-cell-intrinsic alterations. At the invasive front, dissociated tumor cells interact continuously with extracellular matrix, cancer-associated fibroblasts (CAFs), blood and lymphatic vessels, and immune cells. The local microenvironment is therefore an active component of the budding phenotype.

Cancer-associated fibroblasts (CAFs) may create a niche that supports bud survival and migration. Experimental evidence suggests that budding cells can secrete CCL5, recruit fibroblasts through CCR5, and activate angiogenic and collagen-synthesis programs, indicating bidirectional communication between buds and their microenvironment [23].

The contribution of the microenvironment is especially apparent when the invasive front is considered a spatial ecosystem rather than an isolated collection of malignant cells. Experimental observations of bud-derived CCL5 and fibroblast recruitment through CCR5 suggest reciprocal signaling: invasive tumor cells may attract and reprogram fibroblasts, while the resulting matrix and angiogenic changes may support further infiltration [23]. This provides a stronger causal hypothesis than the simpler observation that high budding and desmoplasia frequently coexist, although the extent to which this circuit operates across molecular subtypes and clinical stages remains unresolved.

CMS4 colorectal cancers are enriched in transforming growth factor-β signaling, EMT-associated transcription, angiogenesis, and stromal remodeling; these are biological features that overlap with the invasive-front phenotype. Nevertheless, a CMS4 expression signature derived from bulk tissue can be influenced by the proportion of cancer-associated fibroblasts and other non-neoplastic cells. Spatially resolved measurements are therefore needed before attributing the entire CMS4 signal to the tumor buds themselves [24,25]. The value of integrated pathology may lie in jointly assessing bud density, stromal context, and immune infiltration, not assuming that any one feature is interchangeable with the others [24,25,26].

The extracellular matrix and tumor-cell morphology provide complementary clues to invasive-front biology. A spatially resolved study distinguished true tumor budding from inflammation-associated pseudobudding and identified increased extracellular matrix deposition around true buds [14]. An earlier histopathological study linked cytoplasmic pseudo-fragments around budding foci to aberrant laminin-5 γ2 expression and other features of invasion, without establishing a causal effect [15]. Similar relationships among budding, desmoplastic reaction, and adverse pathological features have been reported in intestinal-type gastric adenocarcinoma [13].

A similar interaction between invasion and immunity has been described in synchronous colorectal liver metastases. In a retrospective multicentre study comprising 107 patients and an external validation cohort of 30 patients, high tertiary lymphoid structure (TLS) density was associated with better survival, whereas extensive budding was associated with poorer outcome. The combined TLS/TB index is promising, although larger independent prospective validation remains necessary [27].

## 7. Tumor Budding Across Histological and Molecular Subtypes

Colorectal cancer is biologically heterogeneous, and the meaning of tumor budding is likely to depend partly on that context. Some histological and molecular backgrounds may favor tumor-cell dissociation, whereas immune-rich microenvironments may modify either its frequency or its prognostic effect.

Serrated adenocarcinoma is a relevant example. Compared with conventional adenocarcinoma, it shows more frequent high-grade budding, cytoplasmic pseudofragments, infiltrative growth, and reduced peritumoral lymphocytic infiltration, suggesting a particularly aggressive invasive front in a subset of serrated tumors [26].

The relationship between budding and mismatch-repair status is less straightforward. Mismatch repair-deficient/microsatellite instability-high tumors often show prominent immune infiltration, yet they do not uniformly lack budding. A retrospective series of 101 patients found no statistically significant difference in budding according to mismatch-repair status assessed by immunohistochemistry [28]. Budding and MSI/MMR therefore provide different, potentially complementary information.

The consensus molecular subtype (CMS) framework provides a biologically plausible but not one-to-one molecular correlate of budding. CMS1 is characterized by microsatellite instability and immune activation; CMS2 by epithelial differentiation and WNT/MYC signaling; CMS3 by epithelial and metabolic features; and CMS4 by mesenchymal, TGF-β-associated, stromal and angiogenic programs [24]. Thus, a failure to find an association between budding and MSI status alone does not adequately test relationships with the CMS classification.

Direct cohort-level evidence supports a particularly strong association with CMS4. In a study of 1320 colorectal cancers from four cohorts, budding counts were higher in CMS4 than in the epithelial CMS2/CMS3 categories, with significant differences across all four cohorts; higher budding was also associated with KRAS/BRAF mutation in the cohorts where mutation data were available [25]. Importantly, the cohorts used different sampling methods (whole-section peritumoral budding versus tissue-microarray intratumoral budding) and staining approaches, limiting direct comparisons of absolute counts and thresholds. The association is robust across settings but does not demonstrate that CMS4 causes budding.

At the regional level, expression profiling of microdissected budding and bulk compartments found an EMT-like budding signature and an apparent shift from a CMS2-like bulk profile to a CMS4-like profile in budding regions [17]. These observations support spatial phenotypic heterogeneity within the same carcinoma, rather than proving an irreversible molecular subtype switch in individual cells. Canonical WNT/β-catenin signaling in CMS2 may provide an epithelial oncogenic background from which invasive-front plasticity emerges, but WNT activation alone should not be equated with extensive budding. The increased CMS4 association most plausibly reflects cooperation between tumor-cell plasticity and a permissive stromal environment [17,24,25].

Clinically, a Bd3 category should not be interpreted as evidence of CMS4 membership; CMS subtype and budding should not be treated as substitutes: one is a molecular classification and the other is an anatomically localized histological measurement. Whether their combination improves calibrated risk prediction over stage, mismatch-repair status, and conventional pathology requires external validation; neither a CMS4 designation nor budding grade currently establishes treatment-specific predictive benefit [24,25].

KRAS, NRAS, BRAF, MSI/MMR, HER2, and NTRK are established biomarkers in specific clinical contexts of colorectal cancer management [29]. Tumor budding does not replace these tests; it provides complementary morphological information about invasive behavior. In a 60-case study, negative HER2 expression was associated with low or intermediate budding, whereas a separate 114-case study found no significant association between LC3β expression and budding [30,31]. These exploratory findings are not sufficiently consistent for clinical use.

To establish clinical added value, future investigations should assess peritumoral budding using a prespecified ITBCC protocol, determine CMS profiles in anatomically matched, clearly annotated regions, and evaluate whether their combination improves discrimination, calibration, and decision-curve performance beyond TNM stage, mismatch-repair status, lymphovascular invasion, and other established covariates. Until such evidence is available, CMS–budding associations support biological interpretation rather than subtype-specific treatment recommendations.

## 8. Overall Prognostic Value in Colorectal Cancer

Across clinical settings, tumor budding consistently identifies an adverse phenotype. Its association with lymphovascular invasion (LVI) is biologically coherent because budding reflects tumor-cell dissociation and stromal invasion, whereas LVI records access to lymphatic or blood vessels. Despite this relationship, the two features are not redundant and should be assessed and reported separately.

In a series of 2652 patients with stage I-III colorectal cancer, LVI was present in 21% and was associated with poorer overall survival, advanced pT category, nodal involvement, mucinous histology, perineural invasion, and budding. Within the LVI-positive subgroup, combined T and N categories separated three prognostic strata with approximately 70%, 52%, and 35% five-year survival [32]. As an observational analysis, these estimates require validation before use as individual patient probabilities.

Recent data also support adverse prognostic value in stage II-III disease [6]. In a cohort of 390 patients with stage II-III colon cancer assessed according to ITBCC, Bd1, Bd2, and Bd3 occurred in 48%, 28%, and 24%, respectively. At 24 months, freedom from recurrence was 92%, 84%, and 69%, and Bd3 independently predicted recurrence (HR 2.39) [33].

These studies extend the prognostic relevance of budding beyond pT1 and stage II disease. However, they do not establish that budding predicts benefit from a particular adjuvant regimen. Its current contribution is therefore best framed as refinement of prognosis within TNM-defined groups rather than selection of a specific systemic therapy [33].

## 9. Tumor Budding in pT1 Colorectal Carcinoma and Superficial Lesions

The management of pT1 colorectal carcinoma is one of the settings in which budding has the most immediate clinical relevance. After endoscopic resection, the practical question is whether surveillance is adequate or whether additional surgery with lymphadenectomy is justified by the estimated risk of nodal metastasis and recurrence.

For practical decision-making after endoscopic resection, Bd2 and Bd3 should be recorded as adverse findings, but neither grade is sufficient by itself to mandate colectomy. Budding must be integrated with unequivocal lymphatic or venous invasion, differentiation, deep and/or extensive submucosal invasion, margin status, and the feasibility of complete pathological assessment. Poorly differentiated clusters may provide complementary evidence of aggressive architecture, but they should not be conflated with ITBCC buds. Fragmented or piecemeal specimens introduce uncertainty about the true deepest invasion, lateral and vertical margins, and the invasive-front hotspot. Such uncertainty should be explicitly documented, rather than assigning apparent low risk on the basis of an incompletely assessable specimen. Multidisciplinary discussion should weigh the combined pathological risk, operative risk, and patient preferences [1,34,35,36,37,38].

Depth of submucosal invasion has traditionally been considered a major risk factor, but its independent contribution is inconsistent across studies. In 264 pT1 carcinomas, several morphometric measures of invasion depth were not independently associated with nodal metastasis; LVI was the only independent predictor. PDCs were associated with nodal metastasis in univariable analysis, whereas the association with budding was borderline (*p* = 0.056) [34].

A practical pathology report should specify the assessment context (untreated resection specimen and peritumoral invasive front), reference method (ITBCC on H&E), counting area (0.785 mm^2^), absolute bud count, and Bd category. If the invasive front is unavailable, the report should state that standard ITBCC peritumoral grading could not be performed; a biopsy-based intratumoral count should not be presented as equivalent. Interpretation should flag substantial inflammation, gland rupture, fragmentation, or treatment effect where these could compromise reliability [1,10,14].

The clinical meaning of Bd2 versus Bd3 is setting-dependent. In pT1 colorectal cancer, Bd2/3 can contribute to a higher-risk assessment after local excision and should be integrated with other established adverse histological factors when discussing additional oncologic resection. For stage II colon cancer, the most consistently supported risk signal is Bd3: it can strengthen recurrence-risk discussions, especially when considered alongside pT category, lymphovascular/perineural invasion, lymph-node yield, and MMR/MSI status. Bd2 should not automatically be given the same weight as Bd3 in stage II disease. Neither Bd category alone selects an adjuvant regimen [1,7,33,34,35,36,37,38,39].

Importantly, prognostic association and treatment prediction answer different questions. A prognostic marker is associated with outcomes, such as lymph node metastasis, recurrence, or survival, irrespective of which therapy a patient receives. A predictive biomarker demonstrates that the relative benefit or response to a specific therapy differs according to the marker, ideally established through a treatment-by-biomarker interaction in appropriately designed studies. Most available budding studies support prognostic utility; retrospective associations between budding and response after chemoradiotherapy should not be described as evidence that budding predicts benefit from any particular regimen. Prospective treatment-interaction evidence remains insufficient for using budding as a stand-alone treatment-selection biomarker [7,9,33,39,40,41].

By contrast, a retrospective model derived from 526 surgically resected submucosal colorectal carcinomas identified lymphatic invasion, budding grade, and depth of submucosal invasion as factors associated with lymph node metastasis. The statistically derived depth cutoff was 2789 μm. For clinical practicality, the investigators also evaluated a rounded threshold of 3000 μm; among tumors without lymphatic invasion and with submucosal invasion below this threshold, the reported lymph node metastasis rate was 1.05% [35]. This estimate is specific to the study-defined subgroup and should not be interpreted as a validated individual risk for all pT1 carcinomas or as evidence that budding can be disregarded. Selection limited to surgically treated patients and the retrospective study design constrain its generalizability. The contrast with studies in which invasion depth loses significance illustrates why depth should be considered alongside budding, lymphovascular invasion, grade, margins, and specimen quality rather than applied as an isolated decision threshold.

A six-factor nomogram developed in 375 patients with early colorectal cancer likewise identified grade, LVI, and budding as independent predictors and achieved an area under the receiver operating characteristic curve of 0.837; the odds ratio for budding was 3.09 [36]. These results are encouraging, although the value of such a model ultimately depends on its performance outside the development cohort.

A 2025 meta-analysis of 57 studies comprising 24,956 patients with pT1 colorectal cancer found that high-grade budding was associated with lymph node metastasis (RR 4.04) and local recurrence (RR 2.35) [37]. The magnitude and consistency of these associations support inclusion of budding in decisions about additional surgery, while heterogeneity in study design and pathology methods limits direct conversion of pooled relative risks into individual probabilities.

Budding may also predict recurrence independently of nodal status. In a cohort of 150 surgically treated pT1 carcinomas, Bd2/3 was independently associated with nodal metastasis and recurrence. Among node-negative patients, five-year recurrence-free survival was 98.7% for Bd1 and 88.2% for Bd2/3; among node-positive patients it was 100% and 37.0%, respectively [38].

Risk models improve stratification but cannot eliminate uncertainty. Rare recurrences have been described after apparently low-risk pT1 lesions, including a 7-mm sigmoid carcinoma with shallow submucosal invasion, no LVI, low budding, and clear margins [42]. Although such cases should not alter management thresholds, they illustrate that histopathological risk assessment is probabilistic rather than deterministic.

## 10. Biopsy Specimens, Rectal Cancer, and Neoadjuvant Treatment

Assessment in biopsy specimens and in rectal cancer treated with neoadjuvant therapy remains less standardized than assessment in conventional resection specimens. ITBCC focuses on peritumoral budding at the invasive front, a region that is often absent from a biopsy. Intratumoral budding may provide useful preoperative information, but it cannot yet be treated as equivalent to standard peritumoral assessment.

Intratumoral budding in pretreatment rectal biopsies has been associated with a lower probability of pathological complete response after neoadjuvant therapy [10]. The finding is clinically interesting, but routine use is limited by biopsy sampling, intratumoral heterogeneity, and the lack of universally accepted biopsy-specific cutoffs.

Post-treatment rectal specimens pose additional challenges. Fibrosis, tumor regression, inflammation, gland fragmentation, and treatment-related atypia may mimic or obscure buds. A systematic review and meta-analysis of 11 studies associated high post-treatment budding with poorer five-year disease-free survival (HR 1.75) and overall survival (HR 1.77), as well as local recurrence and distant metastasis [40].

A subsequent meta-analysis of 1941 patients with locally advanced rectal cancer treated with neoadjuvant chemoradiotherapy reported associations between budding positivity and reduced overall survival (HR 3.24) and disease-free survival (HR 2.54) [41]. However, variation in staining, timing, scoring, and cutoff definitions across the primary studies complicates direct translation into routine practice.

Tumor budding in neoadjuvantly treated rectal cancer therefore appears prognostically informative, but assessment is not yet fully standardized. In the near term, its most credible role is as one component of an integrated pathological evaluation that also includes tumor regression, resection margins, ypTNM stage, and relevant molecular biomarkers. Reproducible criteria for separating viable buds from treatment-related fragmentation remain a practical priority.

## 11. Therapeutic Implications and Clinical Guidelines

Tumor budding should be integrated with other clinicopathological variables rather than applied in isolation. In pT1 carcinoma, it can influence the assessment of whether additional surgery is warranted after endoscopic resection. In stage II disease, it contributes to discussions about recurrence risk and adjuvant therapy. Its role in stage III disease and in neoadjuvantly treated rectal cancer is less firmly established and has not yet translated into consistent treatment-specific recommendations.

The SEOM-GEMCAD-TTD guidelines recognize budding as an adverse pathologic feature in stage II disease, together with T4 category, examination of fewer than 12 lymph nodes, LVI or perineural invasion, obstruction, perforation, and poor differentiation [7]. MSI/MMR status must also be considered: fluoropyrimidine monotherapy is generally not recommended for stage II MSI-high/dMMR tumors in the absence of other indications.

A thoroughly characterized national screening cohort supports the classification of Bd3 as a high-risk group. Among 497 patients with stage II colon cancer, Bd3 was present in 19% and independently predicted time to recurrence (HR 2.617) [39].

Tumor budding should not be used as a stand-alone therapeutic criterion. Bd3 identifies increased recurrence risk in stage II and III disease, but current evidence does not establish that it predicts the magnitude of benefit from adjuvant chemotherapy [33]. In pT1 carcinoma, Bd2/3 has more direct management implications, particularly when it coexists with LVI, poor differentiation, or deep submucosal invasion [34,35,36,37,38].

From an implementation perspective, the interpretation of a budding result must distinguish analytical validity from clinical actionability. A reportable ITBCC grade provides a standardized morphological observation; however, the implications of Bd2 and Bd3 are not interchangeable between a pT1 endoscopic resection and stage II colon cancer. In pT1 disease, the result contributes to a multifactorial assessment of the likelihood of occult nodal disease and the feasibility of additional surgery. In stage II disease, Bd3 primarily contributes to recurrence-risk counselling in conjunction with pathological stage, mismatch-repair status, other adverse features, and patient factors. In neither setting has budding alone been validated as a treatment-benefit biomarker.

The current clinical relevance of tumor budding across the main colorectal cancer settings is summarized in Table 2.

The following practical interpretations are evidence-informed considerations for multidisciplinary discussion, not stand-alone treatment recommendations or substitutes for local clinical guidelines.

Practical interpretation of Table 2 (clinical settings).

pT1 carcinoma: Interpret Bd2/Bd3 alongside lymphovascular invasion, grade, margin status, depth and width of submucosal invasion, and poorly differentiated clusters. Fragmented or piecemeal resections may limit reliable assessment. Consider additional oncologic surgery through multidisciplinary discussion, not from budding alone.

Stage II colon cancer: Bd3 supports a high-risk prognostic discussion together with T4 status, MSI/MMR, examined lymph nodes and other adverse features. It has not been established as a biomarker that predicts benefit from a particular adjuvant chemotherapy regimen.

Stage III colorectal cancer: High budding may refine prognosis but should not independently change chemotherapy selection or duration.

Pretreatment rectal biopsy: Intratumoral budding remains exploratory; peritumoral ITBCC thresholds are not directly transferable to biopsies and should not determine neoadjuvant treatment alone.

Rectal cancer after neoadjuvant therapy: Interpret viable budding with ypTNM stage, regression grade and resection margins, distinguishing it from treatment-related fragmentation.

Metastatic/spatial context: Combined bud–immune or stromal signatures are investigational and require external and prospective validation before treatment use.

## 12. Digital Pathology, Artificial Intelligence, and Prediction Models

Digital pathology and artificial intelligence may reduce observer variability and capture spatial information that is difficult to obtain from manual hotspot counts. Conventional assessment remains sensitive to slide quality, hotspot selection, observer experience, and the distinction between true buds, pseudobuds, and PDCs [3]. Computational methods could automate quantification while making these sources of variability more measurable.

An automated H&E workflow has been developed to delineate the tumor boundary, detect buds, generate density maps, and identify hotspots [43]. A complementary semi-supervised approach using cytokeratin-stained slides achieved high sensitivity and evaluated spatial measures such as bud density and dispersion [44]. Both approaches are technically promising, although performance across different laboratories, scanners, staining protocols, and uncommon morphologies remains a central implementation challenge.

Integrated pT1 models illustrate the potential clinical value of this approach. The International Budding Consortium score combines LVI, budding, depth of invasion, and tumor grade; its discrimination was moderate (AUC 0.68 before and 0.64 after internal validation), so it should be used to support rather than replace multidisciplinary assessment [45]. A narrative review likewise discusses how pathological risk features contribute to prognosis and the interpretation of adjuvant-therapy decisions [46]. A preliminary retrospective spectral CT study (74 patients) explored quantitative imaging parameters for distinguishing Bd1 from Bd2/Bd3 before surgery, but its small size and lack of external validation limit clinical applicability [47]. For clinical adoption, improved discrimination alone is insufficient: models also require external validation, calibration, clinically meaningful thresholds, and evidence of added value beyond simpler approaches.

Immune variables offer another route toward integrated spatial models. In synchronous colorectal liver metastases, a combined TLS/TB index showed stronger prognostic performance than either feature alone [27]. Computational methods may therefore be most informative when they quantify budding within its epithelial, stromal, and immune context rather than simply automate conventional bud counting.

For comparative evaluation of computational budding systems, future studies should report the reference staining method, patient-level separation of training and test sets, external site and scanner validation, performance at clinically relevant Bd1/Bd2/Bd3 thresholds, and the handling of mimics such as fragmented glands and poorly differentiated clusters. Agreement with manual counting is necessary but is not, by itself, evidence that an automated score improves clinical outcomes or changes management appropriately. Claims of treatment selection require independent prospective evidence rather than retrospective model discrimination alone.

## 13. Limitations of the Current Evidence

Several limitations affect the current evidence base. Interobserver variability is most problematic in small samples, inflamed or fragmented areas, pT1 tumors, neoadjuvantly treated specimens, and cases containing pseudobuds or PDCs. ITBCC has substantially reduced the methodological heterogeneity of earlier studies, but newer applications in biopsies, stage III disease, treated rectal cancer, and metastatic resections have not yet reached the same level of standardization.

Tumor budding is primarily a prognostic biomarker. Evidence that it predicts benefit from a specific treatment remains limited, and retrospective associations should not be translated directly into treatment rules. In stage II disease, interpretation should incorporate T4 category, LVI, lymph node yield, MSI/MMR status, comorbidity, and patient preferences. In pT1 carcinoma, budding should be considered together with LVI, grade, margin status, invasion depth, and validated multivariable risk models.

## 14. Future Directions

Future studies should emphasize prospective registration, standardized specimen selection and scoring, blinded central review, and validation across independent cohorts. Future work should determine whether budding changes clinical decision pathways rather than merely improving statistical discrimination in a model.

Computational systems should be evaluated across institutions, scanners, staining protocols, and uncommon morphological patterns, with explicit reporting of calibration, domain-shift performance, failure modes, and clinically relevant decision thresholds. Integrating budding with other invasive-front features, immune variables, molecular biomarkers, and circulating tumor DNA may produce more informative models than any single feature alone.

## 15. Conclusions

Tumor budding is an established morphological biomarker of aggressive behavior in colorectal cancer. Standardized ITBCC assessment has its clearest clinical relevance in pT1 colorectal cancer and stage II colon cancer, with growing prognostic evidence in stage III disease and neoadjuvantly treated rectal cancer. Its value is complementary: budding refines, but does not replace, TNM stage or established pathological and molecular variables. Carefully validated computational methods may further improve reproducibility and allow budding to be interpreted within a quantitative spatial model of invasion, stroma, and antitumor immunity.

## Figures and Tables

**Table 1:** Practical summary of ITBCC-based tumor budding assessment and major diagnostic pitfalls [1].

Element	ITBCC/Recommended Approach	Main Pitfall	Practical Implication
Definition	Single tumor cell or cluster of up to four tumor cells	Confusion with PDCs or fragmented glands	Apply the 1–4-cell definition consistently
Reference stain	Hematoxylin and eosin (H&E)	Cytokeratin may highlight non-bud epithelial fragments	Use immunohistochemistry as an adjunct in difficult cases
Location	Hotspot at the invasive front in resection specimens	Biopsies may not contain the invasive front	Do not directly transpose peritumoral criteria to intratumoral budding
Counting area	Normalize the count to 0.785 mm^2^	Different microscope field areas can alter classification	Document the field normalization used
Grade	Bd1: 0–4; Bd2: 5–9; Bd3: ≥10 buds	Loss of information near category cutoffs	Report both absolute count and Bd category
Diagnostic pitfalls	Exclude pseudobudding, necrotic fragments, and PDCs	Inflammation, rupture, treatment effect, and artifact	Expert review is advisable when management depends on the result

**Table 2:** Current clinical relevance of tumor budding across major colorectal cancer settings.

Clinical Setting	Current Evidence	Potential Use	Key Limitation	References
pT1 colorectal cancer	Strong association with lymph node metastasis and recurrence	Risk refinement after endoscopic resection; supports decisions on additional surgery	Risk remains multivariable and probabilistic	[34,35,36,37,38]
Stage II colon cancer	Bd3 is consistently associated with higher recurrence risk	Prognostic stratification and adjuvant-treatment discussion	Predictive value for chemotherapy benefit is unproven	[7,33,39]
Stage III colorectal cancer	Adverse prognostic association reported	Additional risk refinement	Limited evidence for treatment-specific decisions	[6,33]
Pretreatment rectal biopsy	Intratumoral budding may correlate with response to neoadjuvant therapy	Potential preoperative risk marker	Sampling limitations and non-standardized cutoffs	[10]
Rectal cancer after neoadjuvant therapy	High budding associated with poorer survival and recurrence outcomes	Integrated post-treatment prognostic assessment	Treatment-related fragmentation and methodological heterogeneity	[40,41]
Metastatic/spatial context	Budding may add information when integrated with immune or stromal features	Composite spatial prognostic models	Requires external and prospective validation	[27]

## Data Availability

No new data were created or analyzed in this study. Data sharing is not applicable to this article.
